# Selecting the superior late-leafing genotypes of Persian walnut (*Juglans regia* L.) using morphological and pomological evaluations

**DOI:** 10.1186/s12870-023-04386-6

**Published:** 2023-08-02

**Authors:** Somayeh Soveili, Ali Khadivi

**Affiliations:** Department of Horticultural Sciences, Faculty of Agriculture and Natural Resources, Arak University, Arak, 38156-8-8349 Iran

**Keywords:** Persian walnut, Spring frost, Gene pool, Breeding, Late-leafing

## Abstract

**Background:**

Late-spring frost is one of the major factors limiting and reducing yield of Persian walnut (*Juglans regia* L.) in temperate regions, including Iran. Therefore, in the present study, seedling-originated genotypes of walnut were investigated to identify late-leafing genotypes with high-quality kernels for direct cultivation in orchards or as parents in breeding programs. In the first step, the variation of the selected trees was investigated in terms of traits related to phenology, vegetation, and fruit. In the second step, late-leafing trees were identified and their traits related to kernel quality were investigated to identify superior genotypes.

**Results:**

Strong variabilities were exhibited among the studied genotypes based on the traits recorded. The genotypes showed high variation based on dates of leafing, full male flowering date, and full female flowering date, including very early, early, moderate, and late. After recording the leafing date, 21 late-leaf genotypes were identified and evaluated to select the superiors among them in terms of kernel quantity and quality. Among them, the values of nut-related traits ranged as follows: nut length: 30.12–49.74 mm, nut width: 29.31–37.17 mm, nut weight: 8.77–16.47 g, and shell thickness: 1.15–2.25 mm. The values of kernel-related traits ranged as follows: kernel length: 22.35–35.73 mm, kernel width: 21.79–29.03 mm, kernel weight: 3.22–8.17 g, and kernel percentage: 35.08–53.95%.

**Conclusions:**

According to the ideal values and situations of commercial characteristics of walnut, twelve promising late-leafing genotypes (No. 9, 13, 32, 33, 72, 77, 78, 82, 83, 86, 92, and 98) were identified and are recommended for cultivation in orchards.

## Background

One of the most important nut crops is the Persian walnut (*Juglans regia* L.), which originates from ancient Persia. One of the most important origins and distribution centers of walnuts is Iran, which has a major role in the walnut industry in the world and is also one of the walnut production centers [1].

One of the most important factors limiting and reducing walnut yield in temperate regions, including Iran, is late-spring frost. Active and passive strategies are used by growers to reduce late spring frost damage [2]. The use of late-leafing cultivars is one of these strategies to reduce late spring frost damage [3]. Therefore, the best and most efficient method to reduce or deal with late-spring frost is to pay attention to the genetic potential of walnut, based on which late-leafing cultivars can be identified and introduced, which is a stable and reliable method [4–6].

Bud-break date determines the late-spring frost damage, so that the sensitivity of opened buds to late-spring frost is higher than that of half-opened buds, and the sensitivity of half-opened buds is more than that of dormant buds [7]. In other words, the resistance of flower buds in fruit trees decreases after the buds break. After this stage occurs, if the air temperature drops below zero or near freezing temperature, late-spring frost damage occurs [8]. Therefore, according to the correlation between late-leafing and late-blooming in walnut, finding cultivars with the above characteristics will reduce late-spring frost damage.

The late-spring frost causes damage to walnut orchards every year in temperate regions, including Iran, and the damage is severe in some years. For instance, in 2018, the late-spring frost caused damage to walnut orchards in northern and northeast parts of Iran [9]. Therefore, to solve this problem, it is necessary to introduce late-leafing cultivars [3]. Walnut has been propagated through seeds in Iran for a long time and also has the dichogamous habit, which has increased its genetic diversity [10]. Therefore, the existence of a large genetic diversity of walnut in Iran is of great help to the breeders so that they can identify and introduce superior late-leafing cultivars and genotypes [3]. Therefore, the evaluation of walnut germplasm in Iran is of great importance.

Walnut is of great importance in horticulture and its genetic material is of great interest in conservation strategies and breeding programs [11]. Breeders pay a lot of attention to native walnut genotypes because among them, individuals with suitable and desired characteristics can be found and promising genotypes can be identified [5]. Then, the promising genotypes may be applied to improve the economic characteristics of walnut in the breeding programs [6].

Successful plant breeding programs are highly dependent on their genetic diversity. Investigating and determining genetic diversity is very important. Morphological characteristics are prerequisites for any food product and provide useful information regarding the designing and development of equipment used during various unit operations, such as handling, transportation, sorting, separating, packing, and processing of fruits [5]. Considering that Iran is an important source of walnut populations, evaluating these populations to find genotypes with desired traits such as late-leafing is very important. Therefore, in the present study, seedling-originated genotypes of walnut were investigated to identify late-leafing genotypes with high-quality kernels for cultivation in orchards and also as parents in the breeding programs of walnut.

## Material and methods

### Plant material

In total, 105 genotypes of walnut seedling-originated trees were selected from the Khalajestan area in Qom province, Iran, and were investigated for three consecutive years (2020, 2021, and 2022) with the goal of selection of superior late-leafing genotypes in terms of kernel quantity and quality. The selected genotypes were mature (12–14 years old) and healthy and had a full crop. The Khalajestan area is located at 34˚09′42"N latitude, 50˚06′23"E longitude, and an altitude of 925 m above sea level. The average annual temperature of this area is 18.20 ˚C and its annual rainfall is 148 mm. In the first step, the variation of the selected trees was investigated in terms of traits related to phenology, vegetation, and fruit. In the second step, late-leafing trees were identified and their traits related to kernel quality were investigated to identify superior genotypes. The growth conditions of the selected trees were monitored in terms of nutrition, irrigation, and fighting against pests and diseases and were well managed.

### The characteristics evaluated

In this study, 36 different morphological and pomological characters were examined in the evaluation of the selected genotypes (Table 1). In total, 50 leaves and 50 fruits of each genotype were used to evaluate the traits. A digital scale and an electronic caliper were used to measure traits related to the dimensions and weight of different organs, respectively. The formula kernel weight/fruit weight × 100 was used to calculate kernel percentage. Walnut descriptor [12] was used to estimate qualitative traits (Table 2). The dates of leafing, blooming of female flowers, blooming of male flowers, and harvest for each genotype were recorded. Leafing date was considered when 50% of terminal buds have enlarged and the bud scales have split exposing the green leaves [12]. A control genotype was considered to record the dates of the traits related to phenology so that the earliest leafing genotype was regarded as the control and the leafing date of the rest trees was scored based on it. For the fruit harvest time, the earliest ripened tree was considered as a control, and harvest date of the remaining trees was scored based on that tree.

**Table 1:** Statistical descriptive parameters for morphological traits used to study walnut genotypes

No	Trait	Unit	Min	Max	Mean	SD	CV (%)
V1	Leafing date	Code	1	7	4.54	1.72	37.78
V2	Full male flowering date	Code	1	7	3.27	1.50	45.72
V3	Full female flowering date	Code	1	7	4.30	1.59	37.05
V4	Tree height	Code	1	5	2.87	1.39	48.57
V5	Tree growth habit	Code	1	5	1.55	0.94	60.65
V6	Tree growth vigor	Code	1	5	3.19	1.23	38.40
V7	Leaf color	Code	1	3	2.58	1.02	39.53
V8	Terminal leaflet shape	Code	1	5	3.53	0.93	26.37
V9	Leaf length	mm	28.48	51.11	36.77	3.86	10.50
V10	Leaf width	mm	20.82	33.63	26.38	2.61	9.87
V11	Petiole length	mm	4.75	10.98	7.48	1.09	14.58
V12	Leaflet number	Number	5.00	8.90	6.70	0.81	12.15
V13	Terminal leaflet length	mm	12.96	27.25	16.98	2.09	12.30
V14	Terminal leaflet width	mm	6.76	12.59	9.48	1.16	12.26
V15	Harvest date	Code	1	9	4.24	2.26	53.30
V16	Yield	Code	1	5	3.53	1.50	42.49
V17	Nut shape	Code	1	7	2.18	1.88	86.01
V18	Nut length	mm	29.04	49.74	35.75	3.41	9.54
V19	Nut width	mm	27.57	37.99	31.79	2.37	7.45
V20	Nut weight	g	6.26	16.47	11.52	2.07	18.00
V21	Shell thickness	mm	0.89	2.54	1.49	0.28	19.05
V22	Shell hardness	Code	1	7	3.80	1.53	40.37
V23	Shell texture	Code	1	3	1.80	0.98	54.67
V24	Shell color	Code	1	5	1.84	1.10	59.89
V25	Shell seal	Code	1	5	1.19	0.71	59.50
V26	Shell surface serration	Code	1	3	1.44	0.83	57.71
V27	Kernel length	mm	20.21	35.73	26.87	2.51	9.34
V28	Kernel width	mm	21.17	30.63	25.91	1.98	7.65
V29	Kernel weight	g	2.83	8.17	5.49	1.01	18.49
V30	Kernel color	Code	1	7	2.18	1.99	91.47
V31	Kernel vein	Code	1	5	1.84	1.23	67.07
V32	Ease of kernel removal from nuts	Code	1	5	1.65	1.16	70.30
V33	Kernel filled	Code	1	5	4.31	1.03	23.92
V34	Kernel plumpness	Code	1	5	3.23	1.12	34.67
V35	Kernel shriveling	Code	1	5	1.67	1.03	61.38
V36	Kernel percentage	%	34.41	60.01	47.85	5.10	10.66

**Table 2:** Frequency distribution for the measured qualitative morphological characteristics in the studied walnut genotypes

	Frequency (no. of genotypes)				
Trait	1	3	5	7	9
Leafing date	Very early (8)	Early (29)	Moderate (47)	Late (21)	-
Full male flowering date	Very early (21)	Early (51)	Moderate (31)	Late (2)	-
Full female flowering date	Very early (5)	Early (43)	Moderate (41)	Late (16)	-
Tree height	Low (29)	Moderate (54)	High (22)	-	-
Tree growth habit	Spreading (77)	Upright (27)	Very upright (1)	-	-
Tree growth vigor	Low (15)	Moderate (65)	High (25)	-	-
Leaf color	Light green (23)	Green (82)	-	-	-
Terminal leaflet shape	Wide oval (1)	Oval (75)	Elliptic (29)		-
Harvest date	Very early (20)	Early (30)	Moderate (29)	Late (22)	Very late (4)
Yield	Low (19)	Moderate (39)	High (47)		-
Nut shape	Round (74)	Wide ovate (2)	Ovate (27)	Oval (2)	-
Shell hardness	Paper (10)	Soft (51)	Moderate (36)	Hard (8)	-
Shell texture	Smooth (63)	Moderate (42)	-	-	-
Shell color	Light (64)	Moderate (38)	Dark (3)	-	-
Shell seal	Excellent seal (97)	Slightly open (6)	Moderate (2)	-	-
Shell surface serration	Low (82)	Moderate (23)	-	-	-
Kernel color	Light (74)	Light amber (8)	Amber (15)	Brown (8)	-
Kernel vein	Low (68)	Moderate (30)	High (7)	-	
Ease of kernel removal from nuts	Easy (77)	Moderate (22)	Difficult (6)	-	
Kernel filled	Low (2)	Moderate (32)	High (71)	-	
Kernel plumpness	Low (11)	Moderate (71)	High (23)	-	
Kernel shriveling	Low (72)	Moderate (31)	High (2)	-	

### Statistical analysis

For analysis of variance (ANOVA), SAS software [13] was applied. SPSS software [14] was applied to do Pearson correlation and principal component analyses (PCA). In addition, SPSS software was used for multiple regression analysis (MRA) using stepwise linear method, the purpose of which was to determine the independent traits affecting kernel weight. In MRA, *r*^2^ and *β* coefficients were calculated using regression analysis and were investigated for different traits related to traits. The *r*^2^ coefficient represents the multiple correlation coefficient and measures the correlation between morphological and pomological traits. Also, *β* is the standardized regression coefficient, which is calculated by MRA for each trait-related trait. Besides, the PAST software [15] was used to perform the cluster analysis using Ward’s method and Euclidean distance and to generate a two-dimensional plot based on the first (PC1) and second (PC2) principal components.

## Results and discussion

### Assessment of the 105 genotypes studied

The studied genotypes showed significant differences in terms of measured characters, as revealed using ANOVA (*P* ≤ 0.01). The range of coefficient of variation (CV) values for the studied traits varied from 7.45 (for nut width) to 91.47% (for kernel color) (Table 1). In agreement with the present results, Kavosi and Khadivi [16] reported the lowest CV (8.71%) for nut width and a high CV (78.42%) for kernel color in walnut.

The genotypes showed high variation based on dates of leafing, full male flowering date, and full female flowering date, ranging from very early to late. Tree height was low (29 genotypes), moderate (54), and high (22). Spreading tree growth habit was predominant (77 genotypes) (Table 2).

The range of the leaf length was 28.48–51.11 mm, leaf width was 20.82–33.63 mm, and petiole length was 4.75–10.98 mm. Terminal leaflet shape was predominantly oval (75 genotypes). The range of terminal leaflet length and width was 12.96 -27.25 mm and 6.76–12.59 mm, respectively (Table 1).

Harvest date was highly variable, including very early (20 genotypes), early (30), moderate (29), late (22), and very late (4). Yield was low in 19, moderate in 39, and high in 47 genotypes. Nut shape was predominantly round (74 genotypes). Nut length ranged from 29.04 to 49.74 mm, nut width varied from 27.57 to 37.99 mm, and nut weight varied between 6.26 and 16.47 g (Table 1). Kavosi and Khadivi [16] reported the range of nut length as 26.41–46.94 mm, nut width as 19.58–36.56 mm, and nut weight as 5.18–15.88 g.

Shell was paper (10 genotypes), soft (51), moderate (36), and hard (8). Shell was predominantly excellent (97 genotypes) (Table 2). Shell thickness varied from 0.89 to 2.54 mm. Kavosi and Khadivi [16] reported the range of shell thickness as 0.78–2.98 mm (Table 1).

The values of kernel-related traits ranged as follows: kernel weight: 2.83–8.17 g, kernel length: 20.21–35.73 mm, kernel width: 21.17–30.63 mm, and kernel percentage: 34.41–60.01% (Table 1). Kavosi and Khadivi [16] reported the range of kernel length as 20.32–34.78 mm, kernel width as 12.82–29.12 mm, kernel weight as 1.69–7.52 g with, and kernel percentage as 28.18–59.47%.

**Fig. 1:**
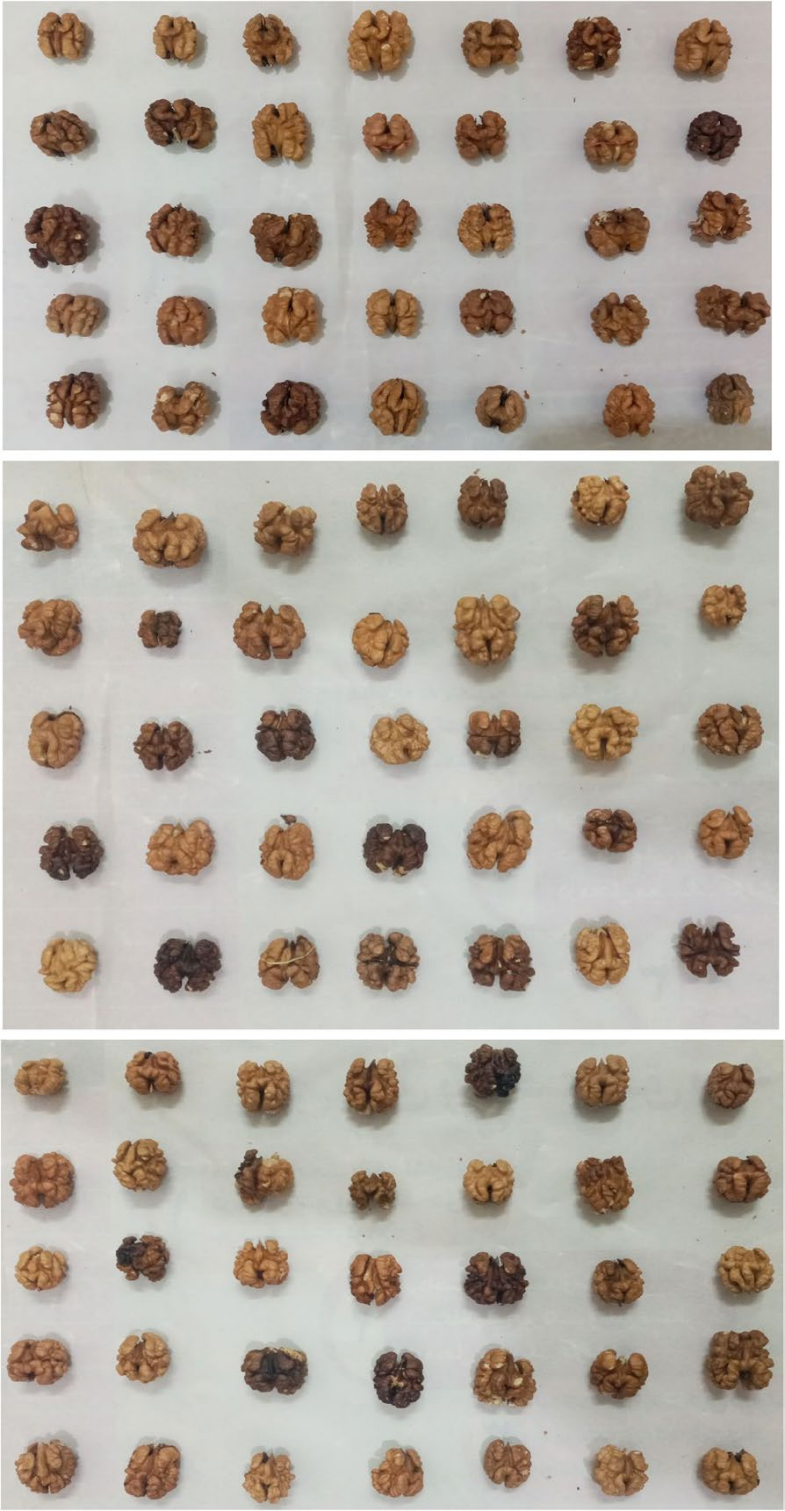
The variation of kernel in terms of size, color, and shape in the walnut genotypes studied

Kernel color in the majority of genotypes (74) was light. Ease of kernel removal from nuts was predominant (77 genotypes) (Table 2). The traits used to study the present germplasm have been previously used in different studies and were confirmed as suitable tools for the evaluation of walnut genotypes [16–20]. The variation of kernel in terms of size, color, and shape in the walnut studied is shown in Fig. 1.

Significant correlations were observed between some quantitative attributes in the studied genotypes (Table 3). Leaf length was highly and positively correlated with leaf width (*r* = 0.65), petiole length (*r* = 0.59), leaflet number (*r* = 0.25), terminal leaflet length (*r* = 0.57), and terminal leaflet width (*r* = 0.40), in line with the previous finding in walnut [16, 19–23]. Nut weight was highly and positively correlated with shell thickness (*r* = 0.27), leaf width (*r* = 0.23), leaf length (*r* = 0.23), terminal leaflet length (*r* = 0.19), nut width (*r* = 0.74), and nut length (*r* = 0.55), in agreement with the previous results in walnut [16, 19–23]. Kernel weight was positively and significantly correlated with leaf length (*r* = 0.29), leaf width (*r* = 0.32), terminal leaflet length (*r* = 0.26), nut length (*r* = 0.54), nut width (*r* = 0.70), nut weight (*r* = 0.83), kernel length (*r* = 0.70), and kernel width (*r* = 0.80), in agreement with the previous results in walnut [16, 19–23].

**Table 3:** Simple correlations between the quantitative morphological variables utilized in the studied walnut genotypes

No	Trait	V9	V10	V11	V12	V13	V14	V18	V19	V20	V21	V27	V28	V29
V9	Leaf length	1												
V10	Leaf width	0.65**	1											
V11	Petiole length	0.59**	0.34**	1										
V12	Leaflet number	0.25**	0.01	-0.33**	1									
V13	Terminal leaflet length	0.57**	0.60**	0.36**	-0.18	1								
V14	Terminal leaflet width	0.40**	0.47**	0.21*	-0.28**	0.50**	1							
V18	Nut length	0.12	0.20*	0.02	-0.03	0.20*	0.14	1						
V19	Nut width	0.23*	0.24*	0.13	0.15	0.16	0.03	0.47**	1					
V20	Nut weight	0.23*	0.23*	0.14	0.04	0.19*	0.17	0.55**	0.74**	1				
V21	Shell thickness	-0.08	-0.03	0.00	-0.19*	0.01	0.19*	-0.03	0.00	0.27**	1			
V27	Kernel length	0.19*	0.25**	0.10	0.00	0.22*	0.11	0.93**	0.53**	0.62**	-0.06	1		
V28	Kernel width	0.30**	0.32**	0.21*	0.10	0.23*	0.10	0.42**	0.89**	0.70**	-0.06	0.55**	1	
V29	Kernel weight	0.29**	0.32**	0.24*	0.04	0.26**	0.13	0.54**	0.70**	0.83**	-0.04	0.70**	0.80**	1

^*^, **. Correlation is significant at *p* ≤ 0.05 and 0.01 levels, respectively

The MRA results (Table 4) showed that six traits, including kernel width, kernel length, nut weight, kernel plumpness, full female flowering date, and kernel filled have significant effects on kernel weight, and thus their fluctuations have a significant effect on increasing or decreasing kernel weight, in line the previous results in walnut [16, 19]. Therefore, breeders should pay attention to the above traits to improve kernel weight in walnut.

**Table 4:** The traits associated with kernel weight in the walnut genotypes studied as revealed using MRA and coefficients

Dependent trait	Independent trait	*r*	*r*^*2*^	*β*	*t*	*p*
Kernel weight	Nut weight	0.838 a	0.70	0.56	8.72	0.00
	Kernel width	0.894 b	0.80	0.36	4.27	0.00
	Nut width	0.914 c	0.84	-0.16	-1.80	0.08
	Shell thickness	0.932 d	0.87	-0.15	-4.21	0.00
	Kernel length	0.940 e	0.88	0.47	4.58	0.00
	Kernel plumpness	0.946 f	0.90	0.10	2.48	0.02
	Nut length	0.950 g	0.90	-0.25	-2.70	0.01
	Full female flowering date	0.953 h	0.91	0.12	3.53	0.00
	Full male flowering date	0.956 i	0.91	-0.08	-2.46	0.02
	Kernel filled	0.958 j	0.92	0.08	2.07	0.04

The PCA showed 11 PCs which contributed 71.79% of the total variance (Table 5). For recorded traits, values more than 0.61 was considered significant. Kernel weight, kernel width, kernel length, nut weight, and nut width showed positive correlations with PC1 and accounted for 12.19% of the total variance. Terminal leaflet width, petiole length, terminal leaflet length, leaf width, and leaf length with positive values, were placed in PC2 with justification of 9.42% of the total variance. Also, PC3 included ease of kernel removal from nuts, shell hardness, and shell thickness and explained 7.07% of the total variance. Therefore, the separation of studied genotypes was mostly influenced by the above traits. Using PCA, traits and genotypes can be classified into different groups, and accordingly, the advancement of breeding programs can be accelerated [16, 19–21].

**Table 5:** Eigenvalues of the principal component axes from the PCA of the morphological characters in the studied walnut genotypes

	Component										
Trait	1	2	3	4	5	6	7	8	9	10	11
Leafing date	-0.02	0.15	0.23	0.64^a^	-0.04	0.06	-0.02	-0.09	-0.30	0.00	-0.05
Full male flowering date	0.09	-0.14	-0.14	0.68^a^	0.02	0.21	-0.10	-0.08	0.05	0.21	-0.13
Full female flowering date	-0.02	0.14	-0.09	0.71^a^	-0.14	-0.14	0.22	-0.07	-0.03	-0.04	-0.12
Tree height	0.19	0.28	-0.02	-0.18	0.06	0.12	-0.09	0.69^a^	-0.14	0.06	0.02
Tree growth habit	0.13	0.12	-0.12	-0.04	0.02	0.03	0.07	0.81^a^	-0.03	-0.06	-0.21
Tree growth vigor	-0.01	-0.09	-0.08	-0.04	0.08	0.03	0.15	-0.15	0.10	0.04	0.82^a^
Leaf color	0.00	0.09	0.13	0.11	0.09	-0.16	-0.01	0.21	0.28	0.62^a^	0.17
Terminal leaflet shape	0.02	0.31	-0.04	-0.05	0.04	0.07	-0.03	0.29	0.14	-0.72^a^	-0.05
Leaf length	0.19	0.77^a^	-0.13	0.08	-0.01	-0.12	-0.13	0.27	0.01	-0.12	0.15
Leaf width	0.17	0.77^a^	-0.07	0.14	0.04	0.08	-0.10	0.17	0.13	-0.07	-0.04
Petiole length	0.15	0.69^a^	-0.06	-0.15	0.08	-0.16	0.05	-0.22	0.00	-0.15	0.02
Leaflet number	0.13	-0.26	-0.19	0.30	-0.12	-0.24	-0.31	0.48	0.10	-0.07	0.27
Terminal leaflet length	0.10	0.77^a^	-0.01	-0.07	0.05	0.12	0.07	0.12	-0.10	-0.03	-0.12
Terminal leaflet width	-0.01	0.68^a^	0.19	-0.04	-0.01	0.21	0.09	0.03	-0.11	0.37	-0.02
Harvest date	0.07	-0.16	0.06	0.72^a^	0.04	-0.01	0.04	0.07	0.03	0.00	0.23
Yield	0.00	0.17	-0.06	0.01	0.03	-0.02	-0.03	0.16	-0.39	0.40	0.54
Nut shape	-0.03	0.04	0.05	0.08	-0.06	0.85^a^	0.07	0.03	-0.05	-0.04	-0.07
Nut length	0.57	0.05	-0.04	-0.03	-0.05	0.73^a^	0.00	0.06	-0.02	-0.11	0.09
Nut width	0.91**	0.08	-0.04	0.07	-0.12	-0.02	0.07	0.09	0.06	0.04	-0.04
Nut weight	0.84**	0.10	0.34	0.01	0.19	0.16	-0.04	0.10	-0.03	0.01	0.01
Shell thickness	0.02	0.07	0.78^a^	-0.05	-0.09	0.07	-0.23	-0.18	-0.09	-0.03	0.00
Shell hardness	-0.04	-0.13	0.81^a^	0.08	0.10	0.05	-0.06	-0.14	0.04	0.12	-0.09
Shell texture	-0.02	0.04	0.05	0.09	-0.02	0.03	0.87^a^	-0.07	0.14	0.13	0.08
Shell color	-0.06	0.00	0.17	-0.02	-0.15	-0.19	0.16	-0.21	0.45	0.15	0.38
Shell seal	-0.11	0.06	-0.03	-0.01	0.04	0.08	0.00	-0.09	0.72^a^	-0.03	-0.01
Shell surface serration	0.13	-0.07	-0.14	0.04	-0.07	0.06	0.85^a^	0.02	-0.06	-0.11	0.10
Kernel length	0.68**	0.10	-0.09	-0.03	0.05	0.61	0.00	0.04	0.05	-0.17	0.10
Kernel width	0.90**	0.19	-0.09	0.07	0.05	-0.09	0.06	0.08	0.05	0.04	-0.04
Kernel weight	0.86**	0.19	0.01	0.01	0.33	0.09	0.03	0.10	0.01	-0.07	0.01
Kernel color	0.19	-0.04	0.01	-0.10	-0.25	-0.23	0.29	0.10	0.59	-0.06	-0.05
Kernel vein	0.30	-0.09	-0.07	-0.09	-0.24	0.01	-0.14	0.02	0.54	0.10	0.04
Ease of kernel removal from nuts	0.06	-0.06	0.85^a^	-0.01	-0.08	-0.12	0.18	0.11	0.03	0.04	0.01
Kernel filled	0.19	0.18	-0.04	-0.14	0.78^a^	-0.04	-0.15	-0.01	-0.10	-0.07	-0.05
Kernel plumpness	0.14	0.09	0.16	0.04	0.80^a^	-0.15	-0.03	0.12	-0.09	-0.09	0.11
Kernel shriveling	0.04	0.14	0.21	0.02	-0.68^a^	-0.12	-0.07	0.06	0.07	-0.24	-0.01
Total	4.27	3.30	2.48	2.16	2.13	2.11	1.98	1.92	1.85	1.50	1.45
% of Variance	12.19	9.42	7.07	6.17	6.07	6.02	5.66	5.48	5.29	4.30	4.13
Cumulative %	12.19	21.61	28.68	34.85	40.93	46.95	52.60	58.08	63.37	67.66	71.79

^a﻿^Eigenvalues ≥ 0.61 are significant

**Fig. 2:**
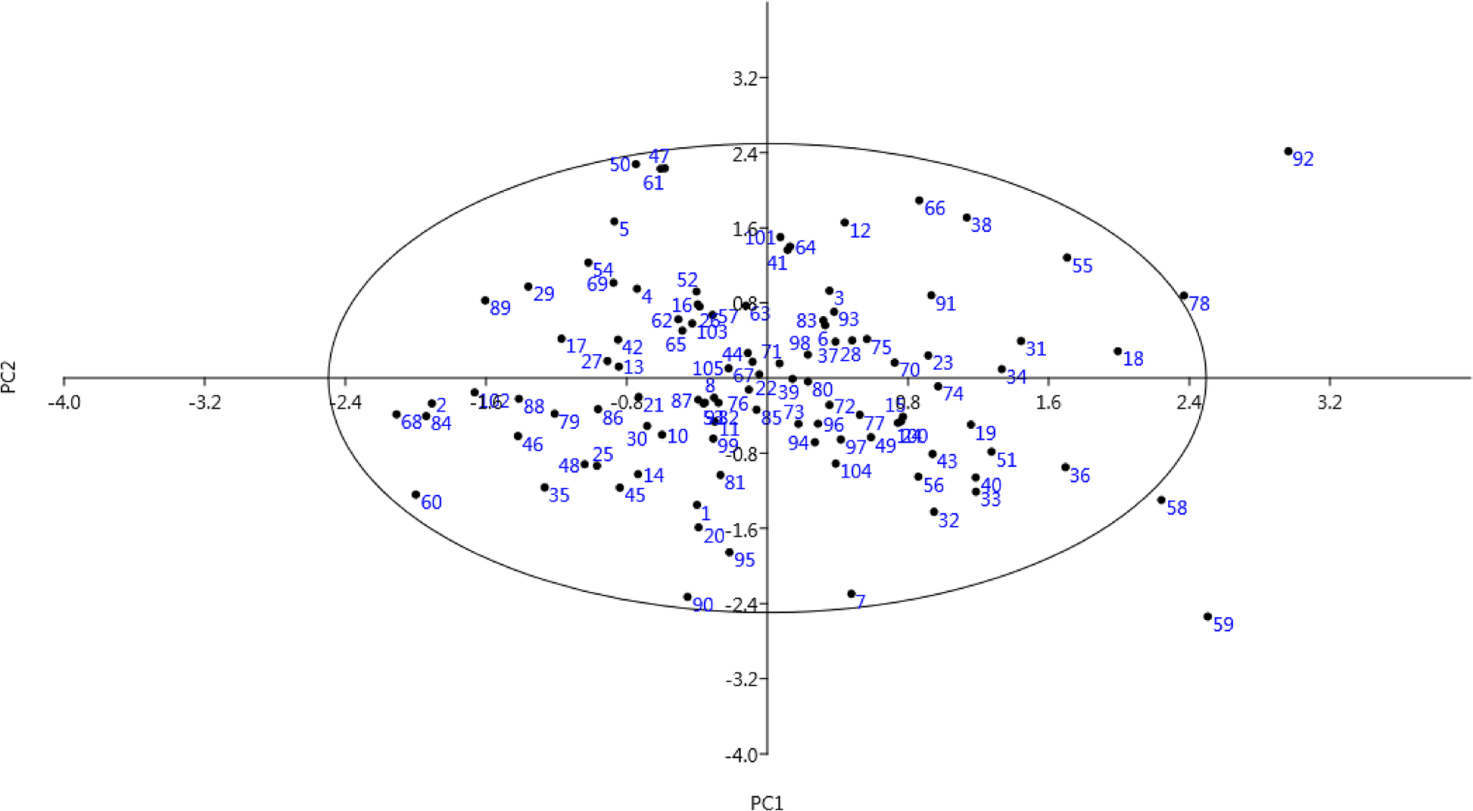
Scatter plot for the studied walnut genotypes based on PC1/PC2

The phenotypic similarities and dissimilarities of the genotypes were analyzed using the bi-plot created according to the attributes placed in PC1 and PC2 (Fig. 2). The genotypes were spread across the surface of the plot, and genotypes 58, 59, 78, and 92 showed the greatest difference with the rest of the genotypes and were placed outside the oval. By starting from negative toward positive values ​​of PC1, a gradual increase in terms of kernel weight, kernel width, kernel length, nut weight, and nut width was observed in the genotypes. Also, by starting from negative toward positive values ​​of PC2, a gradual increase in terminal leaflet width, terminal leaflet length, petiole length, leaf width, and leaf length was observed in the genotypes.

**Fig. 3:**
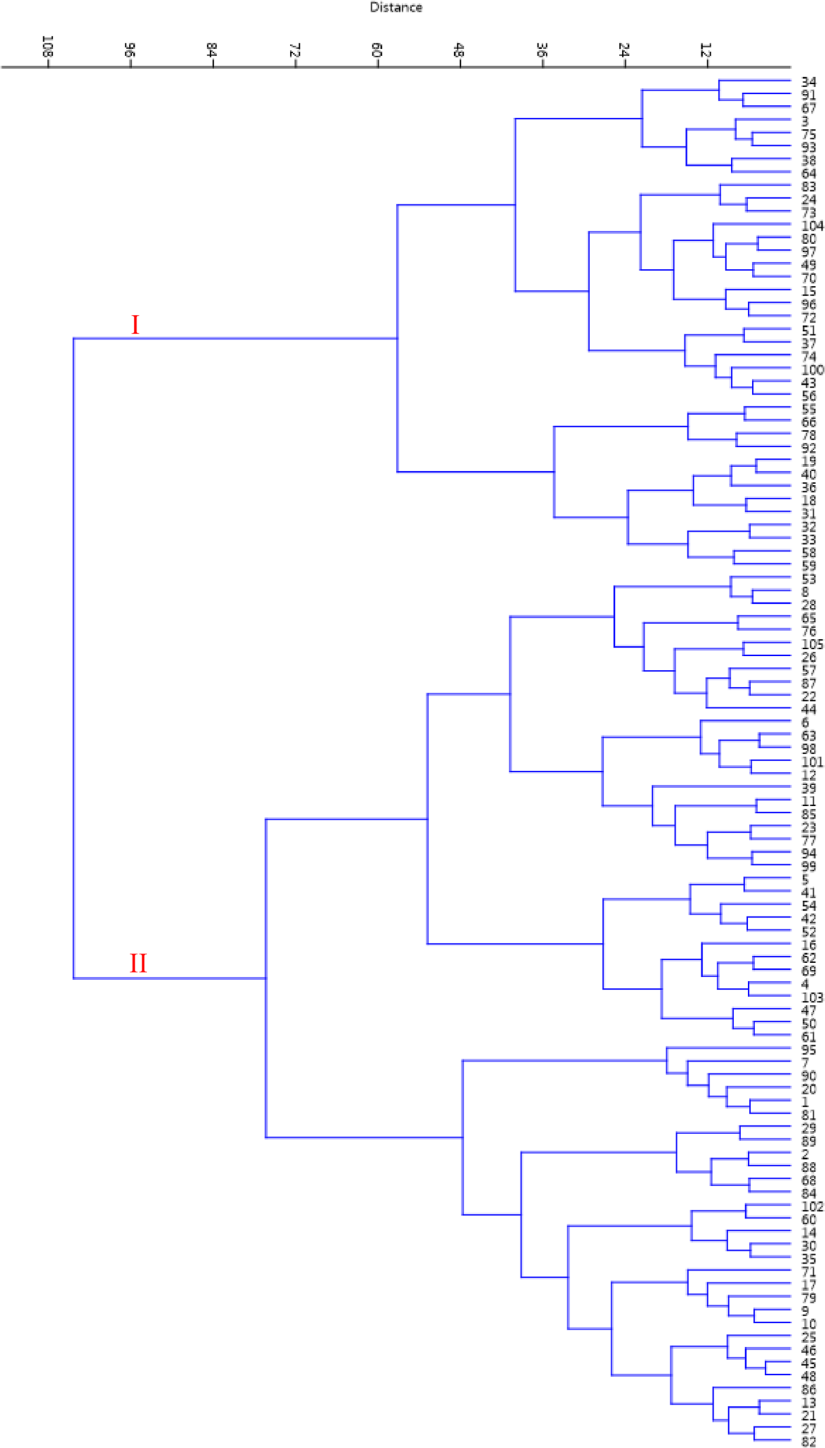
Ward cluster analysis of the studied walnut genotypes based on morphological traits using Euclidean distances

The studied genotypes were classified into two main groups based on Ward's cluster analysis (Fig. 3). The 38 genotypes were placed in the first group (I) with the formation of two subgroups. The second group with 67 genotypes formed two subgroups. The studied genotypes showed strong variations, which could be due to dichogamy, high heterozygosity, and propagation through seeds [24], in agreement with previous findings in walnut [16, 19–23, 25, 26]. Sütyemez et al. [27] studied the phenological differences, genetic diversity, and population structure of Kaman-1 walnut and its 79 progenies and reported a significant variation both phenologically and genetically within the walnut accessions. Also, Bükücü [28] and Sütyemez [29] evaluated phenological differences in walnut genotypes derived from the open-pollinated seeds and reported a wide variation in the studied walnut seedling collection.

### Assessment of the late-leafing genotypes identified

**Fig. 4:**
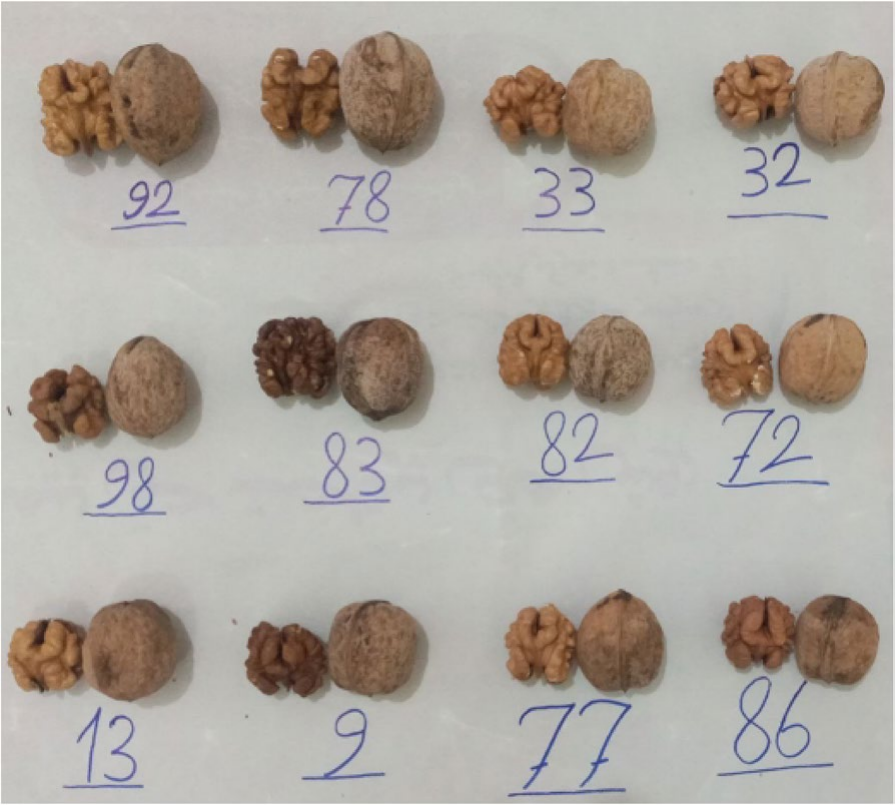
The nuts and kernels of the promising late-leafing walnut genotypes selected

After recording the leafing date, 21 late-leaf genotypes were identified and evaluated to select the promising genotypes among them in terms of kernel quantity and quality. Among them, the CV ranged from 39.57 (in harvest date) to 105.22% (in kernel color). The values of nut-related traits ranged as follows: nut length: 30.12–49.74 mm, nut width: 29.31–37.17 mm, nut weight: 8.77–16.47 g, and shell thickness: 1.15–2.25 mm. The values of kernel-related traits ranged as follows: kernel length: 22.35–35.73 mm, kernel width: 21.79–29.03 mm, kernel weight: 3.22–8.17 g, and kernel percentage: 35.08–53.95% (Table 6). The ideal values for quantitative commercial characteristics of walnut are as follows: nut weight: 12.00–18.00 g [25], shell thickness: 0.70–1.50 mm [30], kernel weight: 6.00–10.00 g, and kernel percentage ≥ 50.00% [31]. Also, the ideal situations for qualitative commercial characteristics of walnut are as follows: paper/soft and well-sealed shells [25], uniform and light kernel color [31], and ease of kernel removal from nut [31]. Thus, according to the ideal values and situations of the above commercial characteristics of walnut, twelve promising late-leafing genotypes (No. 9, 13, 32, 33, 72, 77, 78, 82, 83, 86, 92, and 98) were identified and are recommended for cultivation in orchards. Sütyemez et al. [32] introduced ‘Helete Güneşi’, as a new walnut cultivar with late leafing, early harvest date, and superior nut traits in Turkey. Also, Bükücü [33] studied 74 F1 progenies obtained from ‘Chandler’ × ‘Sutyemez 1’ walnuts in Turkey and reported that the progenies studied are a valuable gene pool for walnut breeding programs. Panahi [34] studied pomological traits related to the fruits of walnut and identified 24 superior genotypes with ideal values that can be cultivated in frost occurrence regions. Khadivi et al. [35] evaluated 67 seed-propagated walnut trees and reported a high diversity in the studied germplasm resulting in the selection of some superior genotypes that can be considered promising plant materials for future walnut breeding programs. The nuts and kernels of the promising late-leafing walnut genotypes selected are shown in Fig. 4.

**Table 6:** Statistical descriptive parameters for morphological traits used to study late-leafing walnut genotypes identified

Trait	Unit	Min	Max	Mean	SD	CV (%)
Harvest date	Code	1	9	5.57	2.20	39.57
Yield	Code	1	5	3.57	1.29	36.05
Nut shape	Code	1	7	2.24	2.14	95.67
Nut length	mm	30.12	49.74	35.28	4.58	13.00
Nut width	mm	29.31	37.17	32.01	1.99	6.22
Nut weight	g	8.77	16.47	11.64	2.10	18.05
Shell thickness	mm	1.15	2.25	1.58	0.30	18.70
Shell hardness	Code	3	7	4.14	1.35	32.66
Shell texture	Code	1	3	1.95	1.02	52.51
Shell color	Code	1	3	1.86	1.01	54.52
Shell seal	Code	1	3	1.19	0.60	50.59
Shell surface serration	Code	1	3	1.48	0.87	58.99
Kernel length	mm	22.35	35.73	26.40	3.13	11.86
Kernel width	mm	21.79	29.03	26.09	1.82	6.97
Kernel weight	g	3.22	8.17	5.45	1.11	20.34
Kernel color	Code	1	7	1.86	1.96	105.22
Kernel vein	Code	1	3	1.38	0.81	58.33
Ease of kernel removal from nuts	Code	1	5	1.76	1.18	66.99
Kernel filled	Code	1	5	4.14	1.20	28.86
Kernel plumpness	Code	1	5	3.10	1.18	38.03
Kernel shriveling	Code	1	5	1.76	1.18	66.99
Kernel percentage	%	35.08	53.95	46.64	4.20	9.00

PCA placed the traits in six components that explained 79.52% of the total variance (Table 7). PC1 accounting for 23.63% of the total variance, was positively and significantly correlated with nut length, nut width, nut weight, kernel length, kernel width, and kernel weight, called fruit size components which exhibited the greatest effect on separating genotypes [36]. Shell hardness, kernel color, and ease of kernel removal from nuts were placed in PC2 and explained 12.56% of the total variance. Shell color, kernel vein, and kernel plumpness were placed in PC3 and explained 12.21% of the total variance. It has been reported that fruit-related traits are important for distinguishing walnut genotypes from each other [19–23, 37].

**Table 7:** Eigenvalues of the principal component axes from the PCA of the morphological characters in the late-leafing walnut genotypes identified

	Component					
Trait	1	2	3	4	5	6
Harvest date	-0.07	-0.09	0.07	0.24	0.00	0.87^a^
Yield	0.45	0.19	0.00	-0.64^a^	-0.20	-0.18
Nut shape	0.34	-0.50	0.02	-0.10	0.59^a^	-0.22
Nut length	0.80^a^	-0.09	0.23	-0.32	0.36	-0.07
Nut width	0.90^a^	-0.02	0.01	0.21	0.11	0.04
Nut weight	0.81^a^	0.07	-0.24	-0.02	-0.05	-0.34
Shell thickness	-0.05	0.37	-0.06	0.31	-0.13	-0.66^a^
Shell hardness	-0.18	0.82^a^	0.05	-0.20	0.00	-0.31
Shell texture	-0.06	0.17	0.08	0.10	0.90^a^	0.28
Shell color	-0.38	0.33	0.68^a^	-0.06	-0.08	0.32
Shell seal	0.01	-0.03	0.45	0.56^a^	-0.15	0.23
Shell surface serration	0.17	-0.03	0.23	0.10	0.77^a^	-0.07
Kernel length	0.87^a^	-0.13	0.16	-0.31	0.19	0.04
Kernel width	0.83^a^	0.02	-0.26	-0.07	0.01	0.32
Kernel weight	0.84^a^	-0.15	-0.36	-0.19	-0.14	-0.05
Kernel color	0.22	0.53^a^	0.34	0.35	-0.01	0.38
Kernel vein	0.08	-0.02	0.73^a^	0.14	0.18	0.04
Ease of kernel removal from nuts	0.05	0.90^a^	0.02	0.21	0.05	-0.15
Kernel filled	0.20	-0.45	-0.52	-0.56^a^	-0.11	0.00
Kernel plumpness	0.28	0.00	-0.74^a^	-0.22	-0.34	0.11
Kernel shriveling	-0.09	0.22	0.18	0.73^a^	0.12	-0.10
Total	4.96	2.64	2.57	2.31	2.20	2.02
% of Variance	23.63	12.56	12.21	11.00	10.49	9.62
Cumulative %	23.63	36.20	48.41	59.41	69.90	79.52

^a﻿^Eigenvalues ≥ 0.53 are significant

**Fig. 5:**
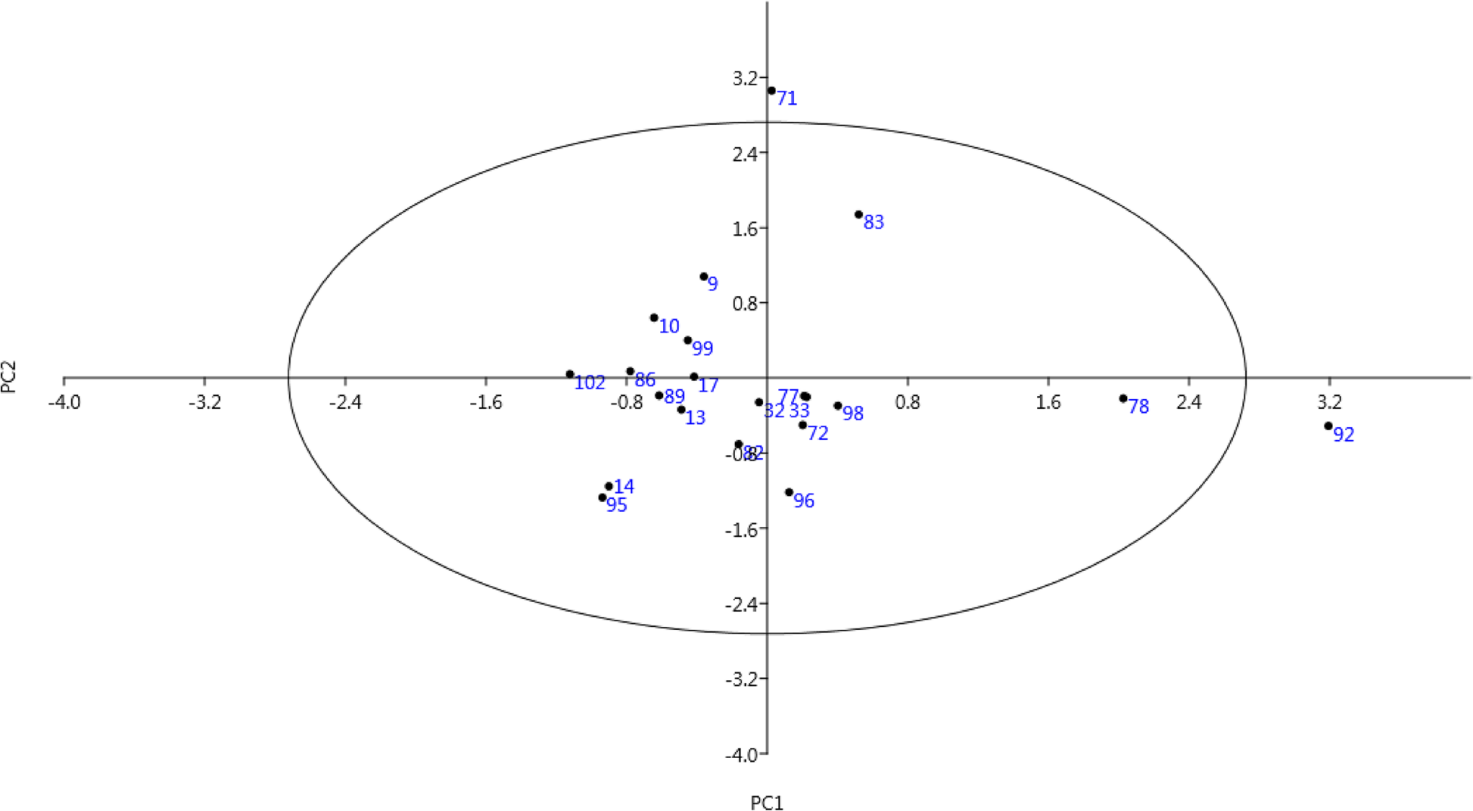
Scatter plot for the late-leafing walnut genotypes based on PC1/PC2

**Fig. 6:**
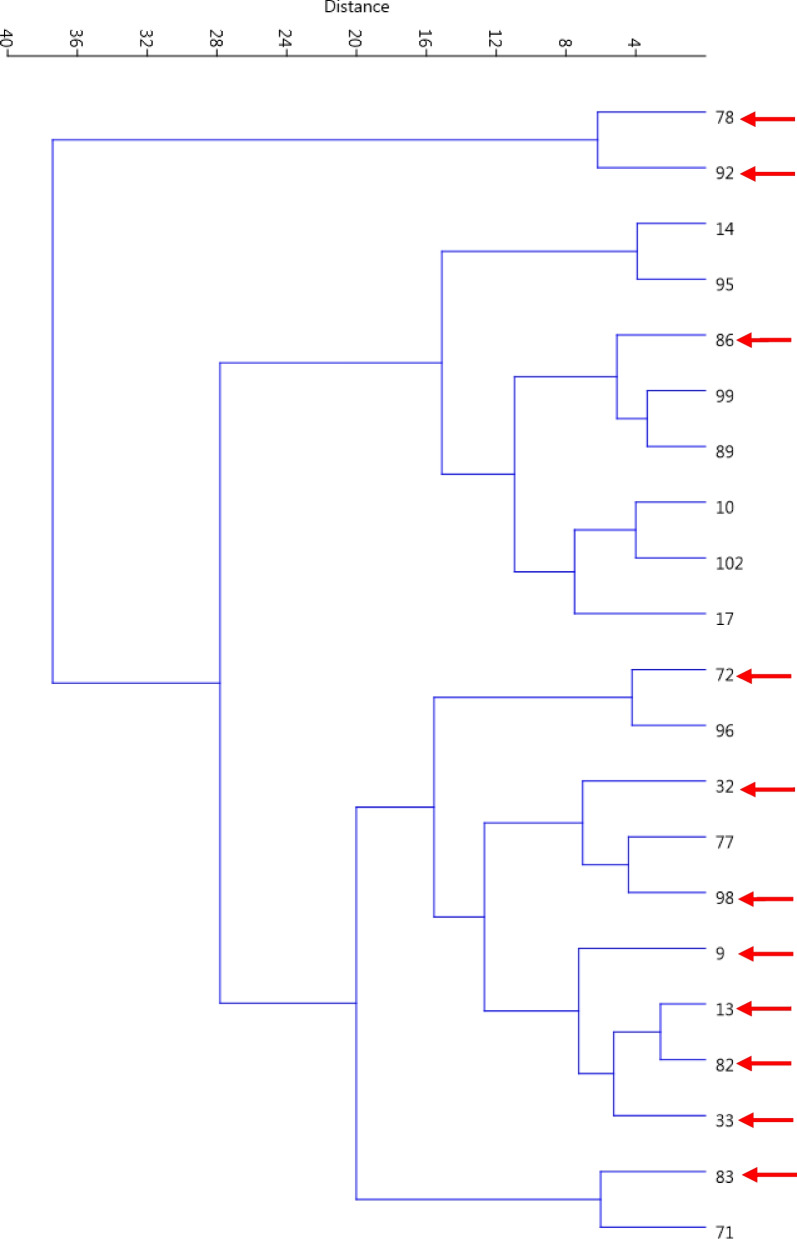
Ward cluster analysis of the late-leafing walnut genotypes based on morphological traits using Euclidean distances. The superior genotypes are marked with arrows

Genotypes were spread across the surface of the plot created based on PC1 and PC2 and showed significant variations, and genotypes 71 and 91 showed the greatest difference with the rest of the genotypes and were placed outside the oval (Fig. 5). The dendrogram created through Ward's method and Euclidean distance placed the genotypes into three groups (Fig. 6). The first group (I) contained 2 genotypes, including no. 92 and 78, characterized by the highest values for nut length, nut width, nut weight, kernel length, kernel width, and kernel weight. The second group (II) consisted of 8 genotypes, characterized by lower values for nut length, nut width, nut weight, kernel length, kernel width, and kernel weight than other groups. The third group (II) consisted of 11 genotypes, characterized by moderate values for nut length, nut width, nut weight, kernel length, kernel width, and kernel weight.

In areas with frequent late-spring frosts, walnut yield is severely reduced. Bükücü et al. [38] investigated genotypic variation and its association with time of leaf budburst and flowering-related traits in 188 walnut accessions and found 16 quantitative trait loci (QTL) with major effects (*R*^2^ between 0.08 and 0.23) to be associated with a minimum of two phenotypic traits each. The present study was carried out in one of the most important areas of walnut production, and then superior late-flowering genotypes were selected to help reduce late-spring frost damage and increase yield.

## Conclusion

One of the major goals of breeding programs in walnut is to identify and introduce superior late-leafing genotypes in terms of kernel quality so that they can escape from the damage of late-spring frost and have high quality from a commercial point of view. Therefore, here, a seedling-originated population of walnut was investigated to achieve the above goal. According to the ideal values of commercial characteristics of walnut, including fruit yield, nut weight, shell hardness, ease of kernel removal from nuts, kernel weight, kernel color, kernel taste, and kernel percentage, 12 late-leafing genotypes, including no. 9, 13, 32, 33, 72, 77, 78, 82, 83, 86, 92, and 98 were promising and may be suggested for cultivation in orchards.

## Data Availability

The data that support the findings of this study are available from the corresponding author upon reasonable request.
